# Artificial intelligence enabled mobile health technologies in arrhythmias-an opinion article on recent findings

**DOI:** 10.3389/fcvm.2025.1548554

**Published:** 2025-02-14

**Authors:** Arijita Banerjee

**Affiliations:** Department of Physiology, IIT Kharagpur, Kharagpur, India

**Keywords:** artificial intelligence (AI), machine learning (ML), atrial fibrillation, supraventricular tachycardia (SVT), mobile health (mHealth), ventricular arrhythmia

## Introduction

Integration of ion channels and transporters and inherent property of automaticity of myocardial cells are necessary for the transmission of electrical impulses throughout the myocardium and the generation of a normal cardiac rhythm. When either of these, normal electrophysiological process of impulse generation or normal conduction of action potential gets disrupted, patients experience cardiac arrhythmias. The risk of acquired arrythmias is significantly increased in presence of structural heart diseases, myocardial infarction and metabolic disorders. The majority of cardiac arrhythmias are categorised according to the rate at which they generate impulses or by where they originate in the myocardium. These include atrial fibrillation (AF), atrial flutter, ventricular tachycardia (VT), supraventricular tachycardia (SVT), ventricular fibrillation and bradyarrythmias (1). With its rapid and erratic electrical signals in the atria, AF is the most prevalent type and causes ineffective contractions. AF patients present with shortness of breath, exhaustion, palpitations, and a higher risk of stroke. Anticoagulation therapy to prevent thromboembolic events along with antiarrhythmic medications, are common management strategies. Sudden cardiac arrest caused by ventricular arrhythmias results in patients losing consciousness. In these situations, immediate cardiopulmonary resuscitation (CPR) and defibrillation are critical for survival (2). Global estimates indicate that cardiac arrhythmias impact nearly 2% of the world's population and are linked to significant socioeconomic burden. According to recent research, machine learning algorithms may enhance the risk stratification for long-term cardiac arrhythmia. The development of mobile health technologies has provided customer-focused health care opportunities (3). In this opinion, the potential applications of the current and upcoming mHealth technologies for treating cardiac arrhythmias are illustrated.

## Role of digital technologies in arrythmia care

Contemporary portable gadgets designed for health monitoring, such as photoplethysmography and ECG systems (4) are not only affordable but the high-speed internet access of these sensors have enabled patients to access healthcare more widely. These technologies enable real-time monitoring and early arrhythmia detection, allowing patients to better manage their conditions and receive timely medical intervention (5). Using longer-term event recorders, 24–48 Holter, or medically certified ambulant electrocardiogram (ECG) monitors has been necessary for the crucial step of correlating symptoms with rhythm. Long-term rhythm monitoring is made possible by mHealth devices, especially smartphone-based ECG and PPG technology, which is reasonably priced (6). Cardiac electrophysiology has been profoundly modified by AI and machine learning, with advancements in mobile technology enabling the measurement of heart-related physiological data. The healthcare industry now has access to a wealth of data, including accelerometers, ECGs, and PPG signals (7).

### Atrial fibrillation screening

New developments in current contact-free plethysmography using smartphone cameras on the face and fingers have demonstrated promise for examining atrial fibrillation. AI algorithms demonstrate a high degree of cardiac rhythm discrimination when used for ECG readings, including those recorded with mobile cardiac telemetry. It will take thorough algorithm validation, data integration with the healthcare system, improvement of current clinical workflows, and strong patient access to turn these fascinating findings that lead to better clinical results expanded to unprecedented levels. Some excellent examples of recent innovations include the TeleCheck-AF project, smartphone ECG surveillance, and home antiarrhythmic medication loading with smartphone tracings (8).

Smartwatches can detect irregular pulses, confirming AF diagnosis through ECG patch monitoring, as demonstrated in studies like Apple Heart Study (9) and Fitbit and Huawei Heart Studies (10). The HEARTLINE trial explores the impact of accessible devices like Apple Watch on early AF detection and clinical outcomes, while the LOOP trial explores anticoagulant use (11, 12). Population-wide AF screening may benefit, but its usability and detection depend on the screening modality and population characteristics. Diagnosis of AF is typically made through ECG or continuous ECG recording, but concerns about false-positive diagnoses arise from sensitivity and specificity variations. The detection rates of new AF have only ranged from 0.9%–7.4% using handheld ECG monitors like Merlin, which rely on automated algorithms and have sensitivities ranging from 93%–100% (13). PPG and ECG-based wearables can identify AF in patients with previous AF, anti-arrhythmic medications, cardioversion, or ablation as highlighted in Table 1. While ECG is still the gold standard for diagnosing AF, PPG is already found in the majority of commercially available smartphones and wearable technology, making it a low-cost way to monitor arrhythmias even if it doesn't allow for precise diagnosis. Further ECG evaluation is necessary to confirm arrhythmias identified by PPG alone.

**Table 1:** Validated devices involving mHealth technologies for arrythmia.

Type of Device	Study	Sensitivity (%)	Specificity (%)	Arrythmia	Pros	Cons
Hand-held ECG devices	Zenicor (14)	96	92	AF	Cloud-based analysis service	No display of ECG tracings
	MyDiagnostick (15)	100	96	AF	Recording and storage device	Rechargeable battery not available
	Merlin ECG event recorders (13)	93.9	90.1	AF	Display of ECG recordings	Rechargeable battery not available
Wearable PPG based	Apple Watch (9)	98	90.2	AF/flutter/SVT	Simple technology.	Blood pressure cannot be measured.
					Easy to carry.	PPG sensor consumes more power.
					FDA approved	Expensive.
	Fitbit (10)	68	98	AF/flutter/SVT	Easy to carry.	Blood pressure cannot be measured.
					FDA approved	Expensive.
Smartphone ECG based	AliveCor Kardia (16)	98	97	AF/flutter	ECG recordings displayed	iphone/android is required
	AliveCor (17)	Alivecor is accurate in measuring QTc interval (*P* < .01)		QTc in sinus rhythm	ECG recordings displayed	iphone/android is required
				Easy to carry		
	AliveCor Kardia (18)	89	91	SVT	ECG recordings displayed	iphone/android is required
					Easy to carry	
					FDA approved	
Pulse wave analysis	Pulse-smart (36)	97	93.5	AF, PVC	Can distinguish between sinus rhythm and irregular abnormal pulse.	Noise and light affect accuracy.
					Easy to use.	
Smartphone PPG based	Cardiio Rhythm (19)	92.9	97.7	AF/flutter	Low cost.	Android application needed
					Automatic beat to beat measurement of HR.	
Wearable ECG based	Necklace-ECG (20)	99.1	98.5	AF	ECG tracings available.	Continuous wearing is required.
					Easy, simple.	
	Samsung Simband 2.0 (21)	98.2	98.1	AF/flutter	Both ECG and PPG recordings are obtained.	Android is necessary
Holter based-continuous monitoring	Zio Patch (22)	96 arrhythmia events detected		AF, SVT, VT	No battery charging required	Prolonged wearing
					FDA approved	

ECG, electrocardiography; PPG, photoplethysmography; AF, atrial fibrillation; PVC, premature ventricular contraction; SVT, supraventricular tachycardia; VT, ventricular tachycardia; QTc, corrected QT interval; HR, heart rate.

### Atrial fibrillation management and the potential risk of stroke

The potential of PPG and ECG-based wearables to identify AF in patients with a history of AF, anti-arrhythmic medication use, cardioversion, and ablation has been confirmed by certain studies using artificial neural network (23). Integrating mobile technologies with a “pill in the pocket” approach could provide benefits like closer monitoring, early antiarrhythmic medication administration, confirmatory validation of symptoms, and avoiding side effects and emergency medical visits (24).

In order to predict paroxysmal AF on ECGs from patients in sinus rhythm, the Mayo Clinic created the first AI-ECG algorithm using almost 650,000 ECGs. Furthermore, the use of AI-ECG for AF estimation was investigated in patients who had an embolic stroke of unknown cause, in which silent underlying AF is often suspected to be the cause (25). AI/ML techniques may also present the chance to stratify patients based on outcomes, like the likelihood of a stroke or the success of cardioversion, in the context of a new AF diagnosis. The ML models outperformed the CHA2DS2-VASc and HATCH scores in predicting the risk for ischemic stroke, but they were less effective than the scores in predicting 6-month AF recurrence, 6-month rhythm control, and pharmacological cardioversion success (26, 27). Regardless of the AF pattern (silent or paroxysmal) or whether the AF burden is low due to automatic cessation of rhythm control techniques, current guidelines advise lifelong anticoagulation based on risk factors.

### Role of mHealth in ventricular and supraventricular arrythmias

SVT is challenging to diagnose due to its unpredictable nature and lack of diagnostic yields. Traditional methods have diagnostic yields ranging from 10% to 50%–60%. mHealth devices offer long-term, affordable rhythm monitoring, effectively diagnosing patients experiencing brief episodes of prolonged palpitations. Smartphone-based single-lead ECGs have a high resolution to distinguish SVT from sinus tachycardia misdiagnosis (89% sensitivity and 91% specificity). However, only 51% of surveyed doctors would proceed with an invasive EP study based on symptomatic, regular tachycardia (18). mHealth devices may mistakenly diagnose palpitations caused by PVCs as AF due to irregular rhythms. Discrimination algorithms could address this issue. Smartphone-based algorithms have successfully distinguished PVCs from sinus rhythm, PACs, and AF with 96% accuracy. A computational algorithm created a feature matrix from QRS attributes from a smartphone-connected ECG device, showing 98.69% PVC recognition accuracy. Smartphones may be helpful in diagnosing ventricular arrhythmias, as evidenced by case reports (28, 29). According to the 2019 guidelines published by the European Society of Cardiology (ESC), mobile recording devices may be required for the diagnosis of supraventricular tachycardias (SVT) due to their ease of use, but validation is necessary. EP-guided ablation, a potential treatment, can be accelerated with the use of smartphone-based one-lead ECG recordings. Unfortunately, quantifying burden is difficult due to the irregular nature of SVTs (30).

### Use of mHealth application for arrythmia care in children

Paediatric and congenital heart populations are quickly adopting mHealth technologies, despite the fact that these tools were created and validated in the adult population. When compared to traditional 12-lead ECGs in children, a few mHealth devices, like the Apple Watch and Alive Cor Kardia Monitor (31), have been evaluated for symptom-rhythm correlation and QT evaluation with high quality data (32). ICDs are effective in saving lives for patients with high risk of sudden cardiac death (SCD), but they don't significantly reduce sudden deaths. Machine learning can develop algorithms to recognize reduced left ventricular function from a 12-lead ECG, which predicts ICD benefit (33). Traditional markers and AI-based markers struggle to improve mortality rates by predicting positive and negative values and identifying modifiable physiological processes. Wearable technology measuring heart rate variability (HRV) can improve general health, but its efficacy is limited due to limited data in controlled settings (34).

## Limitations of mHealth technologies

For various stakeholders, integrating digital health technologies into the treatment of patients with arrhythmias poses a number of challenges. Healthcare providers encounter challenges like a lack of knowledge about the features of the devices, a lack of confidence in their use, and worries about liability. Challenges for patients and consumers include the need for additional testing, socioeconomic disparities that impact access, potential anxiety related to test results, and differing levels of digital literacy. Operational difficulties include continuous charging of devices, cybersecurity threats, data storage problems, insufficient investment in workflows to handle the growing number of devices, and the incorporation of device data into electronic health records. Additionally, the adoption of these technologies in the healthcare landscape is made more difficult by the absence of clear guidance on legal obligations and reimbursement proceedings (35).

## Conclusion

High cardiac rhythm discrimination is demonstrated by various AI algorithms in ECG readings. According to recent research, AI algorithms may enhance the risk stratification for long-term ventricular arrhythmia. However, for widespread clinical results, additional validation, data integration, clinical workflow enhancements, and patient access are required. Data from wearable devices being incorporated into electronic medical records (EMRs) is crucial for efficient clinical review and decision-making. However, clinician time is a significant barrier to this integration. By reducing the need for frequent office visits and intervention, these systems can ensure consistent and effective patient care. Clinical data is essential for confirming device accuracy and determining the effectiveness of interventions based on findings. Progressive automation may be an option, but systems should begin as semi-automated. However, to evaluate the clinical usefulness of machine learning models in enhancing subsequent ventricular rhythm disturbances, additional investigations with bigger sample sizes, robust validity, more varied patient samples, are required.

## References

[1] Tse G. Mechanisms of cardiac arrhythmias. J Arrhythmia. (2016) 32:75–81. 10.1016/j.joa.2015.11.003 PMC4823581 27092186

[2] Wang M Tu X. The genetics and epigenetics of ventricular arrhythmias in patients without structural heart disease. Front Cardiovasc Med. (2022) 9:891399. 10.3389/fcvm.2022.891399 35783865 PMC9240357

[3] Amuthan R Curtis AB. Sex-specific considerations in drug and device therapy of cardiac arrhythmias: JACC focus seminar 6/7. J Am Coll Cardiol. (2022) 79:1519–29. 10.1016/j.jacc.2021.11.066 35422248

[4] Sana F Isselbacher EM Singh JP Heist EK Pathik B Armoundas AA. Wearable devices for ambulatory cardiac monitoring: JACCstate-of-the-art review. J Am Coll Cardiol. (2020) 75:1582–92. 10.1016/j.jacc.2020.01.046 32241375 PMC7316129

[5] Sanders D Ungar L Eskander MA Seto AH. Ambulatory ECG monitoring in the age of smartphones. Cleve Clin J Med. (2019) 86:483–93. 10.3949/ccjm.86a.18123 31291182

[6] Shabaan M Arshid K Yaqub M Jinchao F Zia MS Bojja GR Survey: smartphone-based assessment of cardiovascular diseases using ECG and PPG analysis. BMC Med Inf Decis Mak. (2020) 20:177. 10.1186/s12911-020-01199-7 PMC7392662 32727453

[7] Yan BP Lai WHS Chan CKY Au ACK Freedman B Poh YC High-throughput, contact-free detection of atrial fibrillation from video with deep learning. JAMA Cardiol. (2020) 5:105–7. 10.1001/jamacardio.2019.4004 31774461 PMC6902123

[8] Gawałko M Duncker D Manninger M Velden RMJ Hermans ANL Verhaert DVM The European TeleCheck-AF project on remote app-based management of atrial fibrillation during the COVID-19 pandemic: centre and patient experiences. Europace. (2021) 23:1003–15. 10.1093/europace/euab050 33822029 PMC8083545

[9] Guo Y Wang H Zhang H Liu T Liang Z Xia Y Mobile photoplethysmographic technology to detect atrial fibrillation. J Am Coll Cardiol. (2019) 74:2365–75. 10.1016/j.jacc.2019.08.019 31487545

[10] Lubitz SA Faranesh AZ Selvaggi C Atlas SJ McManus DD Singer DE Detection of atrial fibrillation in a large population using wearable devices: the fitbit heart study. Circulation. (2022) 146:1415–24. 10.1161/CIRCULATIONAHA.122.060291 36148649 PMC9640290

[11] Rizas KD Freyer L Sappler N von Stülpnagel L Spielbichler P Krasniqi A Smartphone-based screening for atrial fibrillation: a pragmatic randomized clinical trial. Nat Med. (2022) 28:1823–30. 10.1038/s41591-022-01979-w 36031651

[12] Svendsen JH Diederichsen SZ Hojberg S Krieger DW Graff C Kronborg C Implantable loop recorder detection of atrial fibrillation to prevent stroke (the LOOP study): a randomised controlled trial. Lancet. (2021) 398:1507–16. 10.1016/S0140-6736(21)01698-6 34469766

[13] Kearley K Selwood M Van den Bruel A Thompson M Mant D Hobbs FR Triage tests for identifying atrial fibrillation in primary care: a diagnostic accuracy study comparing single-lead ECG and modified BP monitors. BMJ Open. (2014) 4(5):e004565. 10.1136/bmjopen-2013-004565 24793250 PMC4025411

[14] Doliwa PS Frykman V Rosenqvist M. Short-term ECG for out of hospital detection of silent atrial fibrillation episodes. Scand Cardiovasc J. (2009) 43(3):163–8. 10.1080/14017430802593435 19096977

[15] Tieleman RG Plantinga Y Rinkes D Bartels GL Posma JL Cator R Validation and clinical use of a novel diagnostic device for screening of atrial fibrillation. Europace. (2014) 16(9):1291–5. 10.1093/europace/euu057 24825766 PMC4149608

[16] Lau JK Lowres N Neubeck L Brieger DB Sy RW Galloway CD Iphone ECG application for community screening to detect silent atrial fibrillation: a novel technology to prevent stroke. Int J Cardiol. (2013) 165(1):193–4. 10.1016/j.ijcard.2013.01.220 23465249

[17] Garabelli P Stavrakis S Albert M Koomson E Parwani P Chohan J Comparison of QT interval readings in normal sinus rhythm between a smartphone heart monitor and a 12-lead ECG for healthy volunteers and inpatients receiving sotalol or dofetilide. J Cardiovasc Electrophysiol. (2016) 27:827–32. 10.1111/jce.12976 27027653

[18] Wegner FK Kochhäuser S Frommeyer G Lange PS Ellermann C Leitz P Prospective blinded evaluation of smartphone based ECG for differentiation of supraventricular tachycardia from inappropriate sinus tachycardia. Clin Res Cardiol. (2021) 110:905–12. 10.1007/s00392-021-01856-5 33961097 PMC8103426

[19] Chan PH Wong CK Poh YC Pun L Leung WW Wong YF Diagnostic performance of a smartphone-based photoplethysmographic application for atrial fibrillation screening in a primary care setting. J Am Heart Assoc. (2016) 5(7):e003428. 10.1161/JAHA.116.003428 27444506 PMC5015379

[20] Santala OE Lipponen JA Jäntti H Rissanen TT Halonen J Kolk I Necklace-embedded electrocardiogram for the detection and diagnosis of atrial fibrillation. Clin Cardiol. (2021) 44:620–6. 10.1002/clc.23580 33629410 PMC8119818

[21] Ding EY Han D Whitcomb C Bashar SK Adaramola O Soni A Accuracy and usability of a novel algorithm for detection of irregular pulse using a smartwatch among older adults: observational study. JMIR Cardio. (2019) 3(1):e13850. 10.2196/13850 31758787 PMC6834225

[22] Barrett PM Komatireddy R Haaser S Topol S Sheard J Encinas J Comparison of 24-hour Holter monitoring with 14-day novel adhesive patch electrocardiographic monitoring. Am J Med. (2014) 127(95):95.e11–17. 10.1016/j.amjmed.2013.10.003 24384108 PMC3882198

[23] Hannun AY Rajpurkar P Haghpanahi M Tison GH Bourn C Turakhia MP Cardiologist-level arrhythmia detection and classification in ambulatory electrocardiograms using a deep neural network. Nat Med. (2019) 25(1):65–9. 10.1038/s41591-018-0268-3 30617320 PMC6784839

[24] Lyth J Svennberg E Bernfort L Aronsson M Frykman V Al-Khalili F Cost-effectiveness of population screening for atrial fibrillation: the STROKESTOP study. Eur Heart J. (2023) 44:196–204. 10.1093/eurheartj/ehac547 36349968 PMC9839418

[25] Attia ZI Noseworthy P Lopez-Jimenez F Asirvatham SJ Deshmukh AJ Gersh BJ An artificial intelligence-enabled ECG algorithm for the identification of patients with atrial fibrillation during sinus rhythm: a retrospective analysis of outcome prediction. Lancet. (2019) 394:861–7. 10.1016/S0140-6736(19)31721-0 31378392

[26] Buck BH Hill MD Quinn FR Butcher KS Menon BK Gulamhusein S Effect of implantable vs prolonged external electrocardiographic monitoring on atrial fibrillation detection in patients with ischemic stroke: the PER DIEM randomized clinical trial. JAMA. (2021) 325:2160–8. 10.1001/jama.2021.6128 34061146 PMC8170545

[27] Bernstein RA Kamel H Granger CB Piccini JP Sethi PP Katz JM Effect of long-term continuous cardiac monitoring vs usual care on detection of atrial fibrillation in patients with stroke attributed to large- or small-vessel disease: the STROKE-AF randomized clinical trial. JAMA. (2021) 325:2169–77. 10.1001/jama.2021.6470 34061145 PMC8170544

[28] Kumar S Banerjee A. Artificial intelligence-enabled smartwatch used for the detection of idiopathic ventricular tachycardia: a case report. Cureus. (2023) 15(7):e42054. 10.7759/cureus.42054 37602056 PMC10432923

[29] Ringwald M Crich A Beysard N. Smart watch recording of ventricular tachycardia: case study. Am J Emerg Med. (2020) 38:849.e3–e845. 10.1016/j.ajem.2019.10.040 31785973

[30] Manninger M Zweiker D Svennberg E Chatzikyriakou S Pavlovic N Zaman JAB Current perspectives on wearable rhythm recordings for clinical decision-making: the wEHRAbles 2 survey. EP Europace. (2021) 23:1106–13. 10.1093/europace/euab064 33842972

[31] Kobel M Kalden P Michaelis A Markel F Mensch S Weidenbach M Accuracy of the apple watch iECG in children with and without congenital heart disease. Pediatr Cardiol. (2022) 43:191–6. 10.1007/s00246-021-02715-w 34468775

[32] Karacan M Celik N Gul EE Akdeniz C. Validation of a smartphone-based electrocardiography in the screening of QT intervals in children. North Clin Istanb. (2018) 6:48–52. 10.14744/nci.2018.44452 PMC6526985 31180383

[33] Kitsiou S Vatani H Paré G Gerber BS Buchholz SW Kansal MM Effectiveness of mobile health technology interventions for patients with heart failure: systematic review and meta-analysis. Can J Cardiol. (2021) 37:1248–59. 10.1016/j.cjca.2021.02.015 33667616

[34] Singh N Moneghetti KJ Christle JW Hadley D Froelicher V Plews D. Heart rate variability: an old metric with new meaning in the era of using mhealth technologies for health and exercise training guidance. Part two: prognosis and training. Arrhythm Electrophysiol Rev. (2018) 7:247–55. 10.15420/aer.2018.30.2 30588312 PMC6304793

[35] Orchard JJ Neubeck L Orchard JW Puranik R Raju H Freedman B ECG-based cardiac screening programs: legal, ethical, and logistical considerations. Heart Rhythm. (2019) 16(10):1584–91. 10.1016/j.hrthm.2019.03.025 30930331

[36] McMANUS DD Chong JW Soni A Saczynski JS Esa N Napolitano C PULSE-SMART: pulse-based arrhythmia discrimination using a novel smartphone application. J Cardiovasc Electrophysiol. (2016) 27(1):51–7. 10.1111/jce.12842 26391728 PMC4768310

